# Mechanical behavior of single-layer ceramized zirconia abutments for dental implant prosthetic rehabilitation

**DOI:** 10.4317/jced.51442

**Published:** 2014-12-01

**Authors:** Manuel Jiménez-Melendo, Oriol Llena-Blasco, August Bruguera, Jaime Llena-Blasco, Rosa-María Yáñez-Vico, Manuel García-Calderón, Cristina Vaquero-Aguilar, Rocío Velázquez-Cayón, José-Luis Gutiérrez-Pérez, Daniel Torres-Lagares

**Affiliations:** 1 Department of Condensed Matter Physics, University of Seville, 41012, Seville, Spain; 2 Private dental practice. Barcelona, Spain; 3 Dental technician. Barcelona, Spain; 4 Department of Stomatology, University of Seville, 41009, Seville, Spain; 5 Virgen del Rocío Hospital, Clinical Management Unit (UGC) Oral and Maxillofacial Surgery, Seville, Spain

## Abstract

Objectives: This study was undertaken to characterize the mechanical response of bare (as-received) and single-layer ceramized zirconia abutments with both internal and external connections that have been developed to enhanced aesthetic restorations. Material and Methods: Sixteen zirconia implant abutments (ZiReal Post®, Biomet 3i, USA) with internal and external connections have been analyzed. Half of the specimens were coated with a 0.5mm-thick layer of a low-fusing fluroapatite ceramic. Mechanical tests were carried out under static (constant cross-head speed of 1mm/min until fracture) and dynamic (between 100 and 400N at a frequency of 1Hz) loading conditions. The failure location was identified by electron microscopy. The removal torque of the retaining screws after testing was also evaluated. Results: The average fracture strength was above 300N for all the abutments, regardless of connection geometry and coating. In most of the cases (94%), failure occurred by abutment fracture. No significant differences were observed either in fatigue behavior and removal torque between the different abutment groups. Conclusions: Mechanical behavior of Zireal zirconia abutments is independent of the type of internal/external connection and the presence/absence of ceramic coating. This may be clinically valuable in dental rehabilitation to improve the aesthetic outcome of zirconia-based dental implant systems.

** Key words:**Dental implant, zirconia, ceramic structure, mechanical properties.

## Introduction

Metal-based [in particular titanium] implant/abutment structures have been considered for a long time the best option for implant-supported dental restoration due to their good mechanical and functional behavior. There is, however, an increasing clinician and patient demand for enhanced [and predictable] aesthetic levels not only in anterior but also in posterior dental restorations. This challenge has led to the rapid development and introduction of different implant-supported ceramic-based dental systems (1-14). Among ceramics, zirconia [ZrO2] is probably the most investigated material in the last twenty years following the discovery of the stress-assisted tetragonal-to-monoclinic transformation in partially-stabilized zirconia alloys. This phase transformation, usually referred as martensitic transformation, is accompanied by a 3-5 % volume expansion, which helps to arrest [or at least to minimize] the propagation of cracks. This unique transformation is therefore a powerful strengthening mechanism which imparts superior strength and fracture toughness to zirconia alloys when compared with traditional ceramics. This material also exhibits an improved biocompatibility even compared to titanium (1-4,15). The minimal requirements for medical applications of zirconia implants are described in the ISO standard No. 13356.

**Figure 1:**
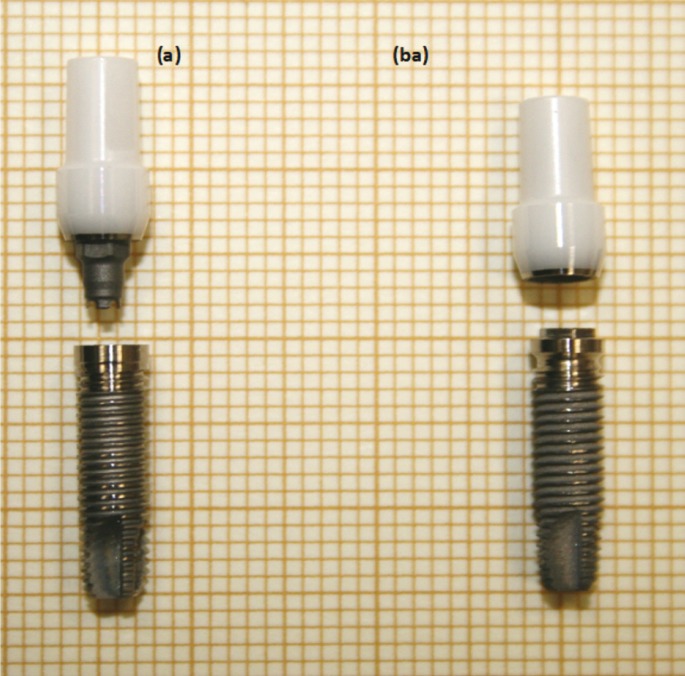
Dental structures used in this study: ZiReal zirconia abutments and Osseotite titanium implants with (a) internal and (ba) external connections.

ZiReal posts belong to a new generation of advanced zirconia implant abutments, (5) with a hex titanium insert fused to the apical end of the abutment. Such a configuration, either with internal or external connection (Fig. 1), enables metal-to-metal contact at the abutment/implant interface, which would result in the same level of precision than all-metal implant systems, avoiding the undesirable effects of abrasion and wearing reported in ceramometal contacts (5,6,16). Furthermore, different aspects of the implant abutment can be customized, as the emergency profile or the prosthetic margin. However, data on the mechanical behavior of this kind of abutments are still very scarce (6,9,12).

Despite their advantages, zirconia abutments do not allow for color control, which can cause aesthetic problems in the case of abutment exposure. Coating the abutment with a ceramic layer of the same color than the eventual prosthetic crown may help to blend in the abutment with the adjacent tissue; this ceramization process is been used successfully with other types of abutments, UCLA for instance. However, the thermal treatment accompanying the coating deposition should not degrade the mechanical performance of the original abutment. Reports about this effect are still lacking.

The objective of this work was therefore to evaluate the mechanical behavior of dental configurations formed by ZiReal zirconia abutments/Osseotite titanium implants under different loading conditions. Static and cycling tests were performed on abutments with internal and external connections, as well as on bare and porcelain single-layer coated abutments. The fracture pattern was also investigated. The structural and microstructural properties of the zirconia abutments studied in this work have been previously characterized and reported elsewhere (17).

## Material and Methods

Sixteen single-tooth implant-abutments systems have been studied in this work. The dental structures consist of (Fig. 1):

[i] Titanium implants [Osseotite®, Biomet 3i, USA] of 4.0 mm in diameter and 15.0 mm in length.

[ii] Cylindrical-shaped zirconia abutments [Zireal Post®, Biomet 3i, USA] of 10.0 mm in length with a performed shoulder 5.0 mm in diameter 3.5 mm from the base, and a neck 4.0 mm in diameter and 0.5 mm thick. A titanium insert is fused at the apical end of the abutment by a proprietary process, providing a metal-to-metal connection with the titanium implant. Abutments with both internal [eight specimens] and external [eight specimens] hex connections were analyzed.

[iii] Titanium hex screws of 8.0 mm in length and 2.0 mm in diameter.

Half of the specimens of each group of connection types were manually coated with a single layer of a low-fusing fluroapatite ceramic [IPS e-max Ceram®, Ivoclar Vivadent Inc., Liechtenstein] of about 0.5 mm in thickness of the same color as the eventual crown. These abutments were fired at 750 ºC for 1 h in air to fix the ceramic layer.

In this way, four subgroups of four abutments each one were obtained: bare [as-received] specimens with internal connection, bare specimens with external connection, coated [ceramized] specimens with internal connection, and coated specimens with external connection. The abutments were placed on the implants and tightened with the titanium screws to 20.0 N.cm using a standard clinical torque gauge [measurement accuracy ± 2.5 N.cm]. Mechanical tests were carried out on the dental structures in air at room temperature on a universal tes-ting machine [Microtest EM 1/50/FR, Spain] equipped with tempered stainless steel push rods. Two types of experiments were performed: static loading tests in compression at a constant cross-speed of 1 mm/min until fracture; and cycling load tests [fatigue tests] between 150 and 400 N at a frequency of 1 Hz with an upper cycle limit of 4x105 cycles. The fracture strength, the origin of fracture and the number of cycles to failure were determined. In order to simulate unfavorable masticatory conditions, the load was applied 30 degrees off the axis of the implant. To this end, an experimental setup was designed and attached to the upper push rod of the deformation machine. A high purity polycrystalline alumina pellet was placed on the top of the lower push rod to prevent any possible indentation. The results obtained for each subset of abutments were analyzed by means of the Mann-Whitney U test and the Kruskal-Wallis H test using SPSS v. 11 software for Windows [LEAD Technologies Inc., USA].

After testing, the specimens were inspected using stereomicroscopy [Leica S8APO; Leica Microsystem, Wetzlar, Germany] and scanning electron microscopy [Philips XL30 [Royal Philips, Amsterdam, The Netherlands], [Microscopy Service, University of Sevilla, Spain] to identify the origin of the failure. The samples were first coated with gold to avoid charging effects during observation. The torque required to unfasten the retaining screws was also measured after the mechanical experiments because screw loosening was reported in the past to be a frequent complication in single-implant restorations (18,19).

## Results

**Figure 2:**
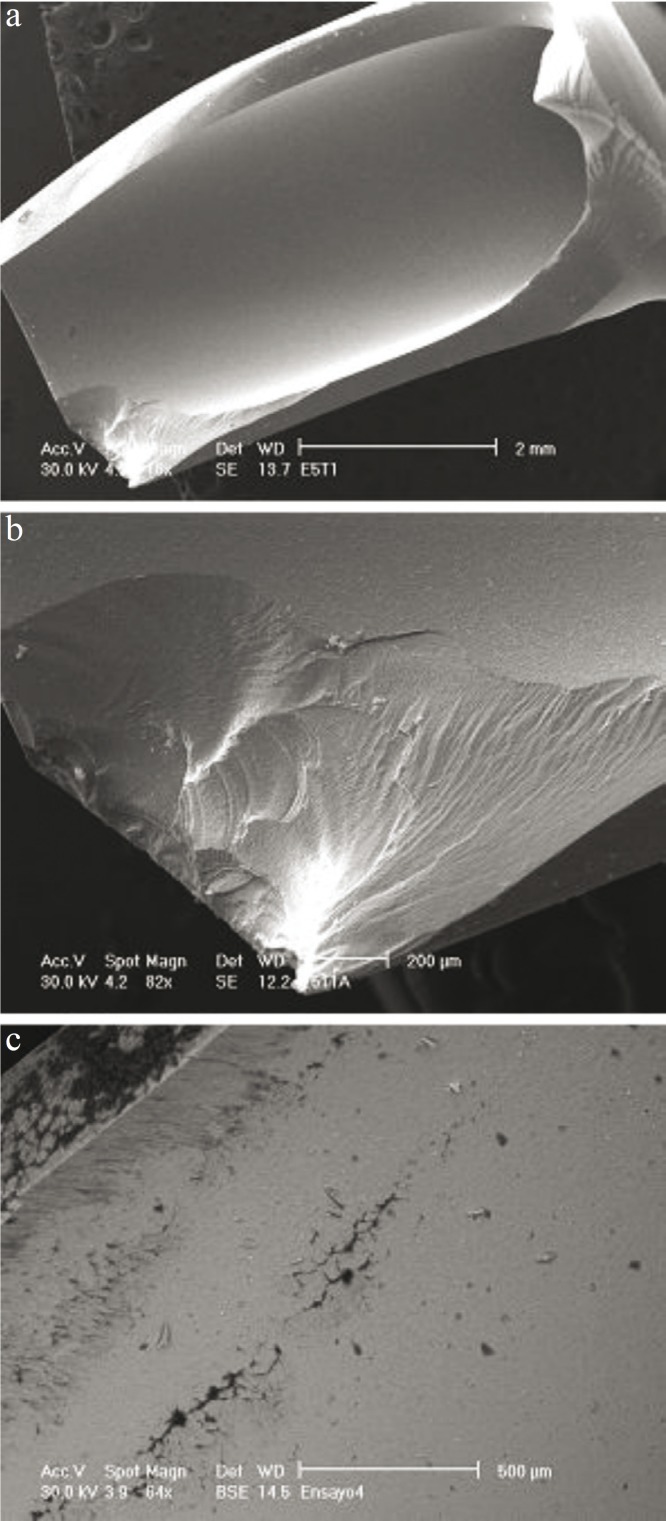
SEM micrographs of a fractured bare abutment after static loading: (a) General view of the critical crack initiated at the point of contact between the abutment and the machine push rod (bottom left); (b) Higher magnification of the fracture initiation area showing hackle lines emanating radially from the crack origin; and (c) Secondary axial cracks developed on the inner surface of the abutment.

**Figure 3:**
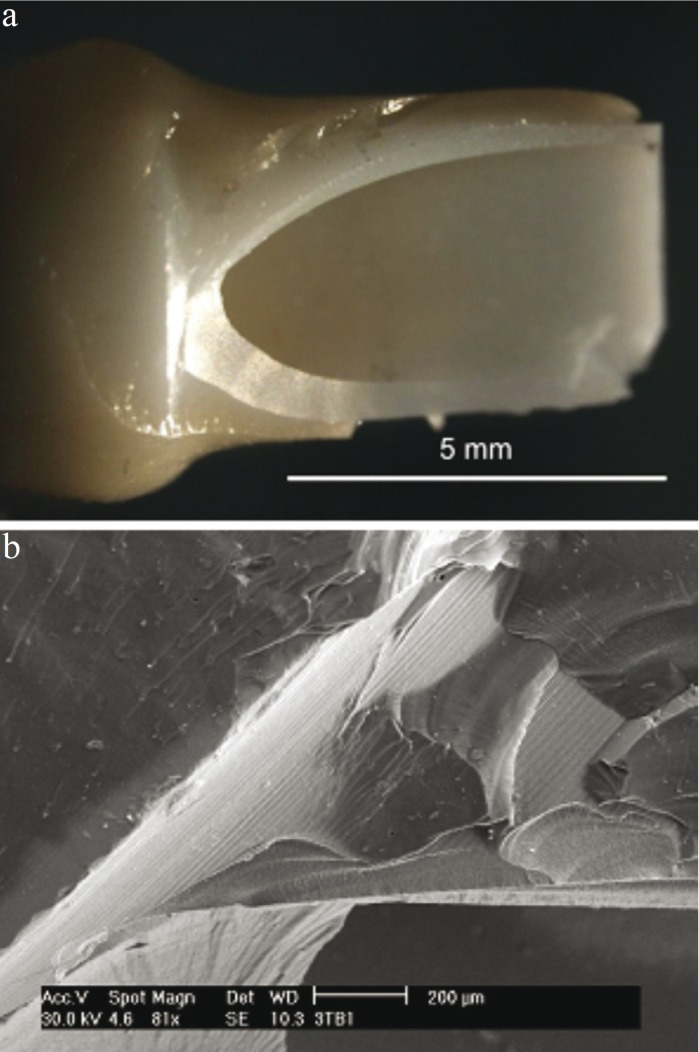
One-layer fluroapatite-coated abutment fractured under static loading: (a) Optical image showing coating loss around the main crack; and (b) Higher magnification SEM micrograph depicting the coating/zirconia interface; milling trace lines can be observed in areas where coating was peeled off.

 Table 1 summarizes the mean values of the various magnitudes measured in the tests: fracture strength, number of survival cycles and removal torque after testing, for as-received and coated specimens, regardless of connection geometry. The average fracture resistance is similar for both groups: 349 ± 37 N for bare abutments and 356 ± 65 N for coated ones. Furthermore, no difference in fracture pattern was observed between both groups. The onset of fracture was systematically located at the point of load application between the abutment and the lower push rod. The critical crack developed from this point axially towards the abutment shoulder, causing the loosening of about half of the abutment (Fig. 2). Hackle lines are visible on the fracture initiation area (Fig. 2); these lines are parallel to the direction of crack advance and usually appear when the crack moves rapidly. Along with the main crack, other secondary axial cracks could be observed on the inner surfaces of the abutments (Fig. 2). The fluroapatite-coated abutments showed some coating loss around the main crack, remaining intact the rest of the abutment (Fig. 3).

**Table 1:**
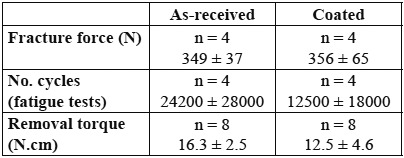
Fracture force, number of cycles to failure and removal torque after testing for as-received and one-layer coated abutments, regardless of connection geometry. No statistically significant differences were detected between both groups.

Regarding the fatigue tests, only one specimen of each group survived the preselected number of 4x105 cycles. The other specimens [75% of the cases] failed prematurely ( Table 1) with the same fracture pattern observed under static loading. Finally, the removal torque values measured for both bare and coated abutment groups are lower than the initial tightening torque ( Table 1); no significant differences, however, were observed in the values of the two groups.

The same information but as a function of the type of abutment/implant connection, internal or external, is gathered in  Table 2. The average values of the fracture strength and removal torque are somewhat higher for the external connection group, though the differences are not statistically significant. For the sake of completeness, the results obtained from a cross-analysis of all abutments are shown in  Table 3. It must be noted that, from the sixteen abutments studied, fifteen failed by abutment fracture as indicated above, while the other one [internal geometry, coated] failed under static loading by detachment of the titanium insert from the zirconia body, remaining intact the rest of the abutment. This specimen, however, failed at a force level of 365 N, which is within the range of fracture strengths exhibited by the rest of specimens. By contrast, the corresponding removal torque was much lower with a value of 5 N.cm. This abutment has been included in the analysis shown in tables 1, 2, 3. If this specimen is excluded [the failure mode is essentially different from the others], the removal torque increases up to 10.0 ± 2.5 N.cm, without significant differences with the other groups.

**Table 2:**
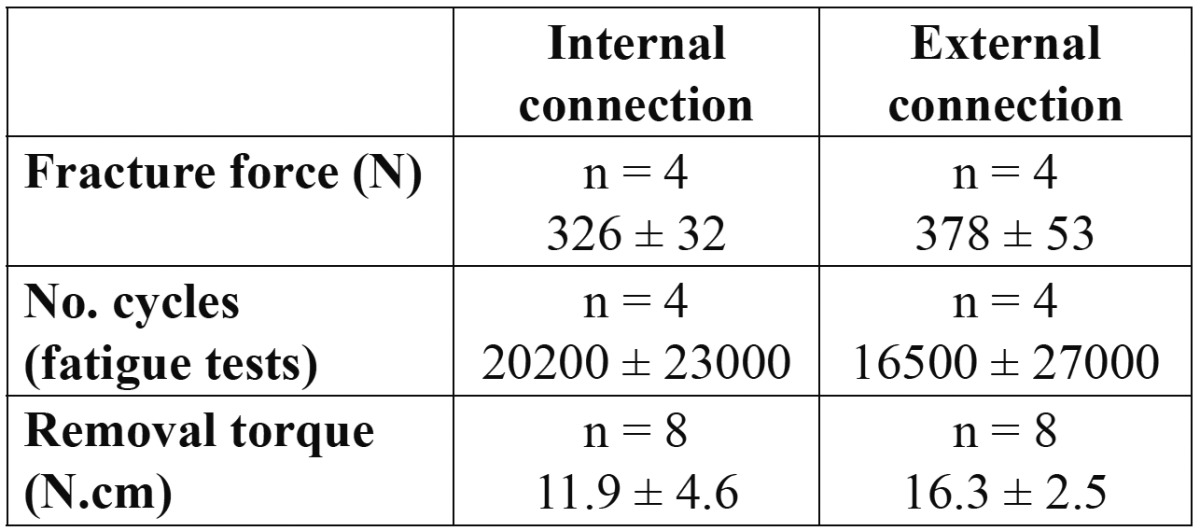
As in table 1 for abutments with internal and external connections, regardless of the presence/absence of coating.

**Table 3:**
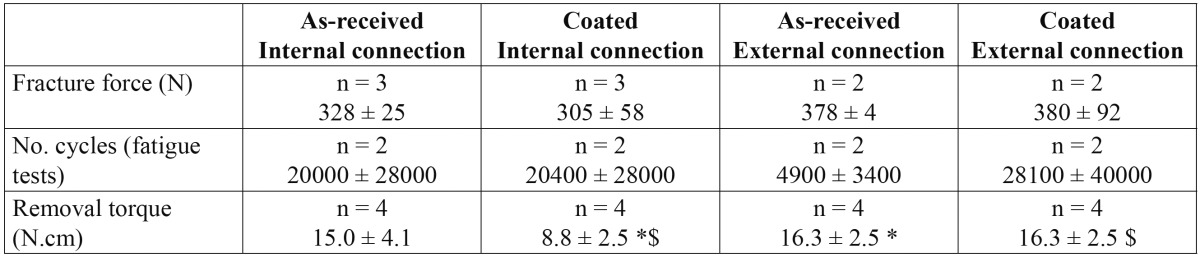
Same magnitudes as in Tables 1 and 2 as a function of the connection type and coating (* p < 0.05; $ p < 0.05).

## Discussion

The structural and microstructural characteristics of the ceramic posts investigated in the present work have been previously reported (17). Briefly, the abutments are formed by 3 mol% yttriadoped zirconia stabilized in the tetragonal phase, and exhibit a very homogeneous and fine grain distribution with an average grain size of 0.30 µm. This microstructure is particularly suitable for the tetragonal-to-monoclinic transformation [the so-called martensitic transformation], which imparts improved strength and fracture toughness to the material. The fracture resistance force results obtained in this study indicate that the different abutments investigated have a similar mechanical strength, regardless of type of connection and coating. This result is especially relevant because it seems that the ceramization process has no detrimental effects on the mechanical response of the dental implants.

Data available in the literature concerning the mechanical behavior of this type of zirconia-based dental systems are still very scarce (6,9,12). Butz et al. (6) reported fracture strengths ranging from 240 to 450 N, with a mean value of 294 N, in the study of sixteen abutments of the same type used in the present study but restored with metal crowns, after exposition to 1.2x106 cycles in a chewing simulator at a frequency of 1.3 Hz and under a mean load of 30 N; the fracture load of the original, non-fatigued abutments was not reported. In that study, failure occurred by abutment fracture in 25% of the cases, by retaining screw fracture in 13% of the cases, and by crown abutment deflection without abutment fracture in the rest of the cases. Interestingly, the same authors observed fracture forces between 180 and 460 N, with a mean value of 324 N, in titanium abutments restored with the same type of crowns. This average strength value is only somewhat higher than that for ceramic abutments, but the dispersion of the data is also larger, contrarily to what would be expected for all-metal prostheses. The same trend has been recently reported by Aramouni *et al.* (7) in the study of ZiReal abutment/3i Certain implant and titanium [UCLA] abutment/3i Certain implant configurations tested at a constant crosshead speed of 1 mm/min. Fracture resistances of 793 ± 123 N and 794 ± 162 N were found for zirconia and titanium abutments, respectively, the data dispersion being again higher for the all-metallic restorations. These results suggest that the finish quality of ceramic abutments is being continuously raised, improving their reliability, and that the martensitic transformation is very effective preventing microcrack propagation. These two studies also revealed that crown fracture is a common cause of endosseous dental implant failure, resulting from translational/rotational movements at the abutment/crown interface. In this regard, the present results suggest that ceramization of zirconia abutments may be a promising alternative.

Although different studies have investigated the bite forces occurring during mastication, there is no general agreement among them. Apart from the anatomical and physiological characteristics of individuals, it has been reported that maximal occlusal forces occur in the area of the first-molar region, with values ranging between 180 and 850 N, decreasing towards 95-250 N for the incisive region (20-22). The fracture strengths obtained in the present study show that the zirconia abutments can tolerate normal occlusal forces in the anterior part of the mouth. In a four-year prospective clinical study on single-tooth implants, Glauser *et al.* (8) also concluded that zirconia abutments offered sufficient stability to support single-tooth implant restoration in the anterior and premolar regions.

Regarding the load cycling tests, it has been estimated that 2.4x105 cycles in vitro under a load of 50 N is equivalent to one year of clinical use (23). In the aforementioned study by Butz *et al.* (6) on ZiReal abutments submitted to fatigue tests, most of the specimens survived after exposition to 1.2x106 cycles under a mean load of 30 N. In the present work, the abutments were submitted to a much higher mean force of 275 N, above the upper limit of 250 N estimated for the incisive region, and at a maximum load of 400 N, higher than the average fracture strength observed under static loading ( Table 1- Table 3). In 75% of the cases, the abutments failed prematurely, with a fracture pattern identical to that observed in static loaded specimens. These results are in agreement with a recent investigation by Mitsias *et al.* (13) on the fatigue behavior of zirconia abutments restored with metal crowns. The authors reported a decrease in reliability with increasing load after exposure to 5x104 cycles, changing from 83% for 175 N to 18% for 300 N and to 0% for 400 N.

On the other hand, the stability of ceramic dental restorations depends on the area of contact between the retaining screw and the abutment. A decrease in detorque values has been observed in the present study, irrespectively of the connection geometry and the presence/absence of coating. By contrast, recent works performed either in vivo (10) and in vitro (6,9) have reported that screw loosening is a rare event in single implant restorations. Discrepancies in the mechanical performance of zirconia abutments with internal/external geometries can be also found in the literature. While Sailer et al. (11) reported a better mechanical response of abutments with internal connectors, Nguyen *et al.* (12) found that Certain ZiReal post/Osseotite NT Certain combinations with internal connection failed by detachment of the metallic insert from the zirconia body [as found in this study], not observed in ZiReal post/Osseotite NT systems with external connection. In this regards, it should be noted that mechanical data coming from different sources are difficult to compare because differences in dental designs and architectures, as well as in experimental setups, can strongly influence the final results. In vivo studies are thus mandatory to assess the clinical performance of these ceramic-based dental systems. Also it is necessary to confirm this information with others abutments of other commercial houses, as well as to be attentive to changes of design to correct the abutment studied in our work.

Within the limitations of this laboratory study of sixteen zirconia Zireal post/titanium Osseotite implant systems [Biomet 3i], it can be concluded that there are no significant differences in mechanical behavior under static and dynamic loading conditions neither between internal and external geometries, nor between bare [as-received] and one-layer ceramized abutments. The average fracture strength is above the maximum bite force reported in the literature for the incisive region. Specimens failed by abutment fracture in 15 cases [= 94%] and by separation of the metallic insert from the zirconia body in 1 case (6%).
